# Acid sphingomyelinase inhibition induces cerebral angiogenesis post-ischemia/reperfusion in an oxidative stress-dependent way and promotes endothelial survival by regulating mitochondrial metabolism

**DOI:** 10.1038/s41419-024-06935-9

**Published:** 2024-09-04

**Authors:** Ayan Mohamud Yusuf, Mina Borbor, Tanja Hussner, Carolin Weghs, Britta Kaltwasser, Matthias Pillath-Eilers, Bernd Walkenfort, Richard Kolesnick, Erich Gulbins, Dirk M. Hermann, Ulf Brockmeier

**Affiliations:** 1 Department of Neurology, University Hospital Essen, University of Duisburg-Essen, Essen, Germany; 2 Imaging Center Essen (Electron Microscopy), University Hospital Essen, University Duisburg-Essen, Essen, Germany; 3 Memorial Sloan Kettering Cancer Center, New York, NY USA; 4 Department of Molecular Biology, University Hospital Essen, University of Duisburg-Essen, Essen, Germany

**Keywords:** Medical research, Translational research

## Abstract

Acid sphingomyelinase (ASM) inhibitors are widely used for the treatment of post-stroke depression. They promote neurological recovery in animal stroke models via neurorestorative effects. In a previous study, we found that antidepressants including amitriptyline, fluoxetine, and desipramine increase cerebral angiogenesis post-ischemia/reperfusion (I/R) in an ASM-dependent way. To elucidate the underlying mechanisms, we investigated the effects of the functional ASM inhibitor amitriptyline in two models of I/R injury, that is, in human cerebral microvascular endothelial hCMEC/D3 cells exposed to oxygen-glucose deprivation and in mice exposed to middle cerebral artery occlusion (MCAO). In addition to our earlier studies, we now show that amitriptyline increased mitochondrial reactive oxygen species (ROS) formation in hCMEC/D3 cells and increased ROS formation in the vascular compartment of MCAO mice. ROS formation was instrumental for amitriptyline’s angiogenic effects. ROS formation did not result in excessive endothelial injury. Instead, amitriptyline induced a profound metabolic reprogramming of endothelial cells that comprised reduced endothelial proliferation, reduced mitochondrial energy metabolism, reduced endoplasmic reticulum stress, increased autophagy/mitophagy, stimulation of antioxidant responses and inhibition of apoptotic cell death. Specifically, the antioxidant heme oxygenase-1, which was upregulated by amitriptyline, mediated amitriptyline’s angiogenic effects. Thus, heme oxygenase-1 knockdown severely compromised angiogenesis and abolished amitriptyline’s angiogenic responses. Our data demonstrate that ASM inhibition reregulates a complex network of metabolic and mitochondrial responses post-I/R that contribute to cerebral angiogenesis without compromising endothelial survival.

## Introduction

Ischemic stroke is the leading cause of long-term disability and the second leading cause of death [1]. Besides recombinant tissue-plasminogen activator-induced thrombolysis, there are no efficient drugs available that promote stroke outcome [2, 3]. Thus, the development of new therapeutics is of paramount importance. A promising target for stroke treatment is the brain microvasculature composed of cerebral endothelial cells, which form a tight barrier separating the vessel lumen from the brain parenchymal tissue. Since this blood-brain barrier controls the brain microenvironment, microvascular injury has detrimental consequences for stroke recovery [4]. While necrosis due to a lack of energy is the predominant type of cell injury in the ischemic core area, a substantial percentage of ischemic cells after reperfusion die as a consequence of active (partly apoptotic) cell death mechanisms [5]. This ischemia/reperfusion (I/R) injury is due to the re-introduced oxygen that induces reactive oxygen species (ROS) formation in reperfused tissue, causing damage to cellular lipids, proteins, and DNA [6]. Of note, the vascular endothelium was identified as a primary site of ROS generation and major target of injury [7]. When successfully coping with the I/R stressor, brain microvessels are able to sprout and reestablish their barrier functions, thus contributing to successful recovery of the damaged brain tissue [8].

Acid sphingomyelinase (ASM) is a membrane-associated enzyme catalyzing the hydrolysis of sphingomyelin to ceramide, which has central roles in organizing membrane microdomains [9]. Antidepressants, including the tricyclic antidepressant amitriptyline (Ami) and the serotonin reuptake inhibitor fluoxetine, are functional ASM inhibitors, which are commonly prescribed for the treatment of post-stroke depressive disorder [10]. Although previous studies suggested that antidepressants might exert their antidepressant action via noradrenaline or serotonin reuptake inhibitor actions [11], more recent studies found that by far most, if not all, antidepressants have potent ASM inhibitory activity that are instrumental for their antidepressant activities in animal depression models [12]. In a previous work, we found that the antidepressants Ami, fluoxetine, and desipramine promote cerebral microvascular angiogenesis in vitro and in vivo post-I/R in an ASM-dependent way via release of small extracellular vesicles (EVs) from endothelial cells [10]. Besides the observation that ASM inhibitors can trigger oxidative stress [13], the current literature does not provide a comprehensive picture of involved cellular response mechanisms, in particular not for brain endothelial cells.

In order to investigate the cellular responses mediating angiogenesis, we exposed human cerebral microvascular endothelial cells belonging to the hCMEC/D3 cell line to transient oxygen-glucose deprivation (OGD), an in vitro model of I/R injury, and studied effects of the ASM inhibitor Ami on oxidative stress responses. We corroborated our findings in a widely used ischemic stroke model in vivo, that is, transient intraluminal middle cerebral artery occlusion (MCAO) in mice treated with Ami or fluoxetine. By pharmacologically and genetically manipulating cellular stress responses, we found that ASM inhibition promotes cerebral angiogenesis in a ROS-associated, heme oxygenase (HO)-1-dependent way. Of note, cerebral angiogenesis was not induced at the expense of compromised endothelial survival. ASM inhibition induced a complex network of cellular metabolic responses that involved reduced cell proliferation, reduced mitochondrial energy metabolism, reduced endoplasmic reticulum (ER) stress, increased autophagy/ mitophagy, and increased antioxidant responses, which protected against cell injury and death.

## Results

### ASM inhibitor Ami induces endothelial tube formation in normoxia and post-I/R in a ROS-dependent way

**Fig. 1:**
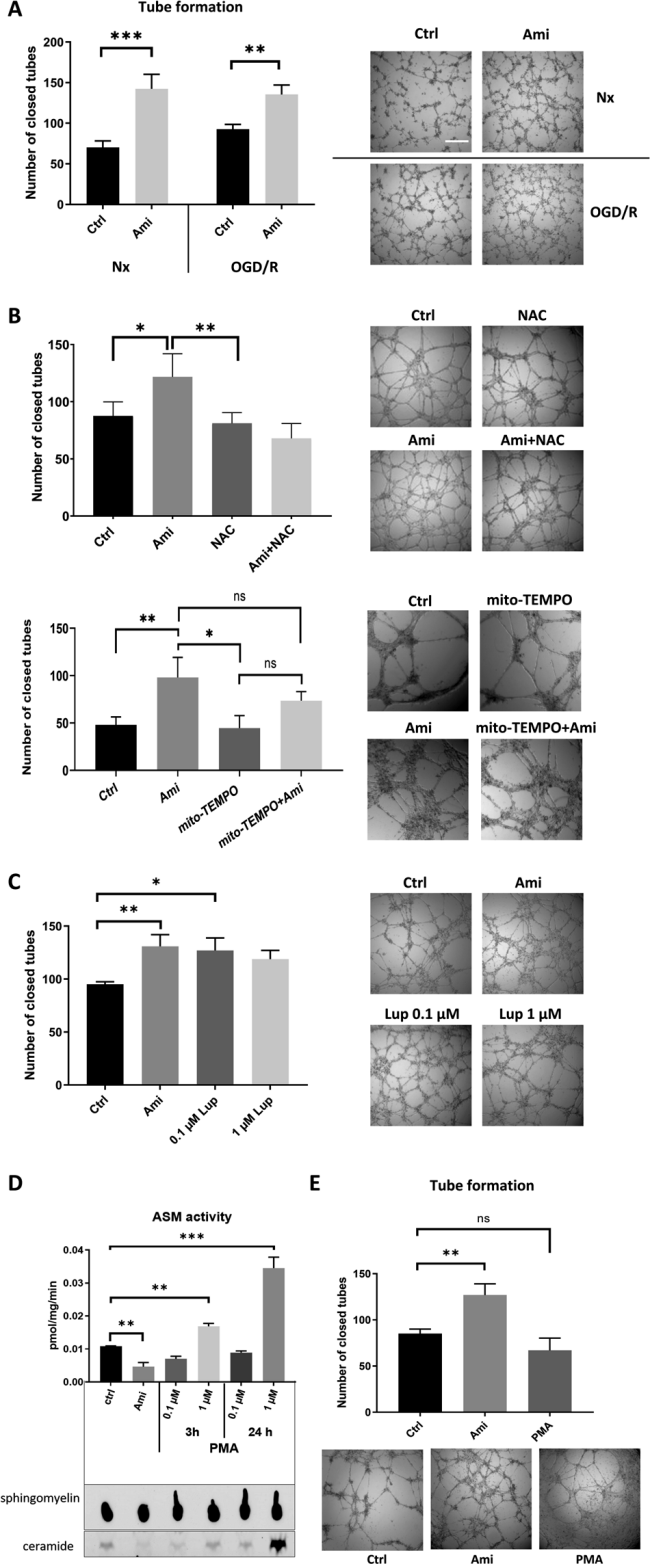
ASM inhibitor amitriptyline induces cerebral angiogenesis in normoxia and post-ischemia/ reperfusion (I/R) in a reactive oxygen species (ROS)-dependent way. Matrigel-based tube formation of microvascular endothelial hCMEC/D3 exposed to normoxia (Nx; 21% O_2_; in **A**–**C**) or oxygen-glucose deprivation (OGD; 1% O_2_; in **A**) for 24 h followed by 3 h of reoxygenation (Reox; 21% O_2_) in full medium (OGD/R), which were treated with (**A**) control medium (Ctrl) or amitriptyline (Ami; 50 µM), **B** Ctrl, Ami (50 µM), the ROS scavenger N-acetyl-L-cystein (NAC; 1 µM), Ami (50 µM) combined with NAC (1 µM), mitochondrial ROS scavenger mito-TEMPO (50 µM) or Ami (50 µM) combined with mito-TEMPO (50 µM) (**C**) Ctrl, Ami (50 µM) or the ROS inducer Luperox^®^ (LUP, 0.1 or 1 µM). **D** ASM activity measured by BODIPY-labeled sphingomyelin of normoxic hCMEC/D3 exposed to Ctrl, Ami (50 µM) or the ASM activator phorbol-12-myristate-13-acetate (PMA; 0.1 or 1 µM) for 3 or 24 h. **E** Matrigel-based tube formation of normoxic hCMEC/D3 cells treated with Ctrl, Ami (50 µM) or PMA (1 µM) for 24 h. Samples were analyzed using IMAGEJ software. Representative samples are shown. Scale bar in representative tube formation photographs: 500 µm. Data are mean ± SD values. Evaluated by one-way ANOVA followed by Bonferroni tests. *p ≤ 0.05/**p ≤ 0.01/***p ≤ 0.001 (if not otherwise indicated compared with corresponding Ctrl; n = 4 independent samples/group).

In an earlier work, we demonstrated that Ami increases angiogenesis in vitro and after MCAO in vivo in an ASM-dependent way [10]. Since the cellular mechanisms remained unknown, we exposed cerebral endothelial hCMEC/D3 cells to normoxia (Nx) or OGD (24 h) followed by reoxygenation (Reox; OGD/R) and examined the effect of Ami on tube formation in our well-defined angiogenesis assay [10]. Our previous experiments have indicated that a concentration of Ami ≥25 µM most effectively induced angiogenesis of hCMEC/D3 cells [10]. Thus, throughout this study, 50 µM Ami were used if not otherwise stated. Indeed, Ami treatment increased tube formation of endothelial hCMEC/D3 cells exposed to Nx and OGD/R (Fig. 1A). According to a former study, Ami increases cellular ROS formation in patients with major depressive disorder [13]. Since ROS formation is a known trigger for angiogenesis in vitro and in vivo [14], we manipulated ROS levels by administering the ROS scavenger N-acetyl-L-cysteine (NAC) or the ROS inducer Luperox^®^: NAC reversed the Ami-induced increase of tubes (Fig. 1B), whereas Luperox^®^ similar to Ami increased the tube number (Fig. 1C). We next applied the mitochondria-targeted antioxidant Mito-TEMPO to evaluate the role of mitochondrial ROS in Ami-induced angiogenesis. Despite scavenged mitochondrial ROS, Ami still increased the tube number, albeit to a lesser extent, suggesting that Ami promotes angiogenesis not solely dependent on ROS produced by mitochondria (Fig. 1B). To further assess how the angiogenic effects of Ami are associated with ASM activity, we next evaluated the effect of the ASM activator PMA on tube formation. At a first set of studies, we confirmed that PMA indeed stimulated ASM enzymatic activity (Fig. 1D). In subsequent studies, we found that PMA reduced the tube number compared to the control, albeit these results were statistically not significant (Fig. 1E).

**Fig. 2:**
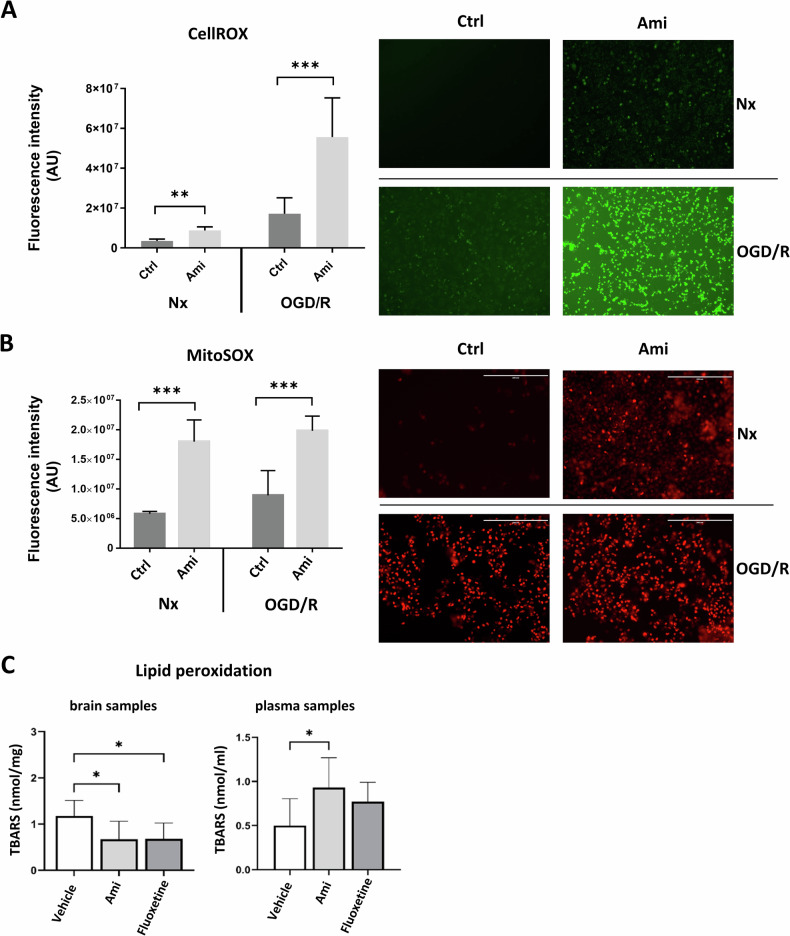
Amitriptyline induces cerebral endothelial intracellular ROS production in normoxia and post-I/R. Oxidative stress, determined by **A** the intracellular ROS detection agent CellROX^®^, a green fluorescent indicator, or **B** the mitochondria-selective ROS detection agent MitoSOX^®^, a red fluorescent indicator, of cerebral microvascular endothelial hCMEC/D3 exposed to Nx (21% O_2_) or OGD (1% O_2_) for 24 h followed by 3 h of reoxygenation (Reox) in full medium for 24 h (OGD/R), which were treated with control medium (Ctrl) or amitriptyline (Ami; 50 µM) during Reox. **C** Lipid peroxidation, evaluated by thiobarbituric acid reactive substances (TBARS) formation, of ischemic brain tissue samples and plasma samples of mice exposed to intraluminal middle cerebral artery occlusion (MCAO), which were i.p. treated with vehicle or the ASM inhibitors Ami (12 mg/kg) or fluoxetine (10 mg/kg) starting immediately after reperfusion. Mice were sacrificed 24 h post-MCAO. Samples were analyzed by ImageJ software. Representative samples are depicted. Data are mean ± SD values. Evaluated by one-way ANOVA followed by Bonferroni (**A**, **B**) or LSD (**C**) tests. *p ≤ 0.05/**p ≤ 0.01/***p ≤ 0.001 (**A** n = 6; **B** n = 4; and **C** n = 5–6 independent samples/group).

To further explore the increased intracellular ROS levels induced by Ami, we used the ROS detection reagent CellROX^®^ due to its ability to get oxidized by a broad range of reactive oxygen species (i.e., superoxide anions, hydroxyl radicals, hydrogen peroxide) and due to its stable emission of green fluorescence light. We noticed that Ami increased the intracellular ROS signal both in Nx and OGD/R cells (Fig. 2A). As CellROX^®^ cannot provide information about the exact origin of intracellular ROS, we also examined the mitochondria-specific ROS indicator MitoSOX^®^. Due to its positive charge, MitoSOX^®^ accumulates preferably in mitochondria and gets oxidized exclusively by superoxide which is the most relevant source of mitochondrial ROS [15]. Clearly, treatment with Ami increased the mitochondrial ROS signal (Fig. 2B) and thus identified the mitochondrial compartment as a site of Ami-induced ROS production.

To investigate if these observations were also relevant in an in vivo model of I/R, lipid peroxidation was measured in peripheral blood samples and reperfused brain parenchyma of MCAO mice treated with vehicle, amitriptyline or the serotonin reuptake inhibitor fluoxetine. Whereas lipid peroxidation in brain parenchyma was reduced by Ami and fluoxetine, which was indicative of reduced ROS formation, lipid peroxidation in blood samples was elevated by Ami and also slightly by fluoxetine (Fig. 2C). Our results indicate that ASM inhibitors increase ROS production in the vascular compartment, whereas the brain parenchyma seemed to be protected against oxidative stress, perhaps by compensatory antioxidant responses.

### Ami reduces mitochondrial energy metabolism post-I/R in cerebral endothelial cells

**Fig. 3:**
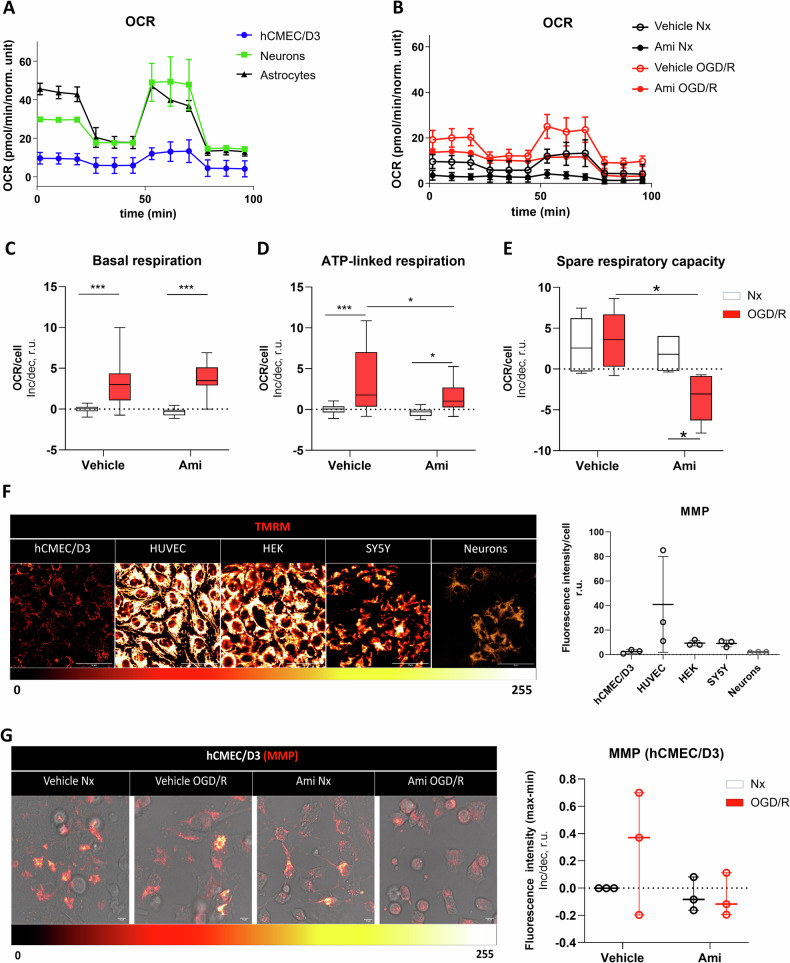
Amitriptyline reduces cerebral endothelial mitochondrial energy metabolism post-I/R. Real-time oxygen consumption rate (OCR), evaluated by Seahorse Mito stress assay before and after administration of the ATP synthase inhibitor oligomycin (for assessing ATP-linked mitochondrial respiration), the uncoupling agent carbonyl cyanide-4-(trifluoromethoxy) phenylhydrazone (FCCP; for assessing spare respiratory capacity), and the complex I and III inhibitors rotenone and actinomycin A (Rot/AA; for distinguishing mitochondrial from non-mitochondrial respiration), of **A** cerebral endothelial hCMEC/D3 cells (in blue), primary astrocytes (in black) and primary neurons (green), and **B** cerebral endothelial hCMEC/D3 cells exposed to Nx (21% O_2_) or OGD (1% O_2_) for 24 h followed by reoxygenation (Reox) in full medium for 2 h (OGD/R), which were treated with control medium (Ctrl) or amitriptyline (Ami; 50 µM) during Reox. OCR values were divided by cell numbers (evaluated by Hoechst staining) in each well and multiplied by a factor of 1000, which defined normalized units. **C** Basal respiration, **D** ATP-linked respiration, and **E** spare respiratory capacity of Nx and OGD/R cells treated with Ctrl or Ami (50 µM). Data are presented as normalized differences (r.u.) to the mean of Nx Ctrl cells. **F**, **G** Mitochondrial membrane potential (MMP) assessed by tetramethylrhodamine-methyl ester (TMRM; in red, phase contrast in gray) of hCMEC/D3, HUVEC, HEK, and SY5Y cells as well as primary neurons exposed to Nx (in **F**) or HCMEC/D3 cells exposed to Nx or OGD/R, which were treated with Ctrl or Ami (50 µM) (in **G**). Representative images are shown. Intensity calibration bar (in **F**, **G**) indicates black as least intense and yellow/white as most intense area. Samples were analyzed by ImageJ software. Data are mean ± SD values (in **A**, **B**) or box plots (medians with interquartile ranges [IQR], with minimum and maximum values as whiskers; in **C**–**G**). Evaluated by two-way ANOVA followed by Bonferroni tests. *p ≤ 0.05/**p ≤ 0.005/***p ≤ 0.0005 (n = 3 independent samples/group).

To elucidate the consequences of oxidative stress on mitochondrial oxidative phosphorylation (OXPHOS), we evaluated electron transport chain (ETC) activity using the Seahorse Mito stress assay (Fig. 3). Comparisons of different types of brain cells revealed that cerebral endothelial hCMEC/D3 cells had significantly lower mitochondrial respiration than primary astrocytes or neurons (Fig. 3A). Basal respiration, ATP-linked respiration, and spare respiratory capacity increased in response to OGD/R (Fig. 3B). Notably, Ami treatment reversed the ATP-linked respiration and spare respiratory capacity induced by OGD/R (Fig. 3B–E). As a complementary approach, we measured mitochondrial membrane potential (MMP) by incubating cells with tetramethylrhodamine-methyl ester (TMRM) for 24 h. Compared to human umbilical vein endothelial cells (HUVECs; which are non-cerebral endothelial cells), SY5Y neuroblastoma cells (which are non-endothelial cerebral cells) and human embryonic kidney (HEK) cells (which are non-endothelial non-cerebral cells), which are widely used in mitochondrial activity studies, hCMEC/D3 cells had a markedly lower MMP similar to primary neurons (Fig. 3F). OGD/R increased MMP in hCMEC/D3 cells (Fig. 3G). Ami reversed this elevated MMP in OGD/R cells (Fig. 3G). Since MMP is a measure of mitochondrial ETC activity [16], these data aligned with findings of the Seahorse Mito stress assay that the ASM inhibitor reduces mitochondrial activity selectively under OGD/R conditions.

### Ami reduces endothelial proliferation and apoptosis post-I/R

**Fig. 4:**
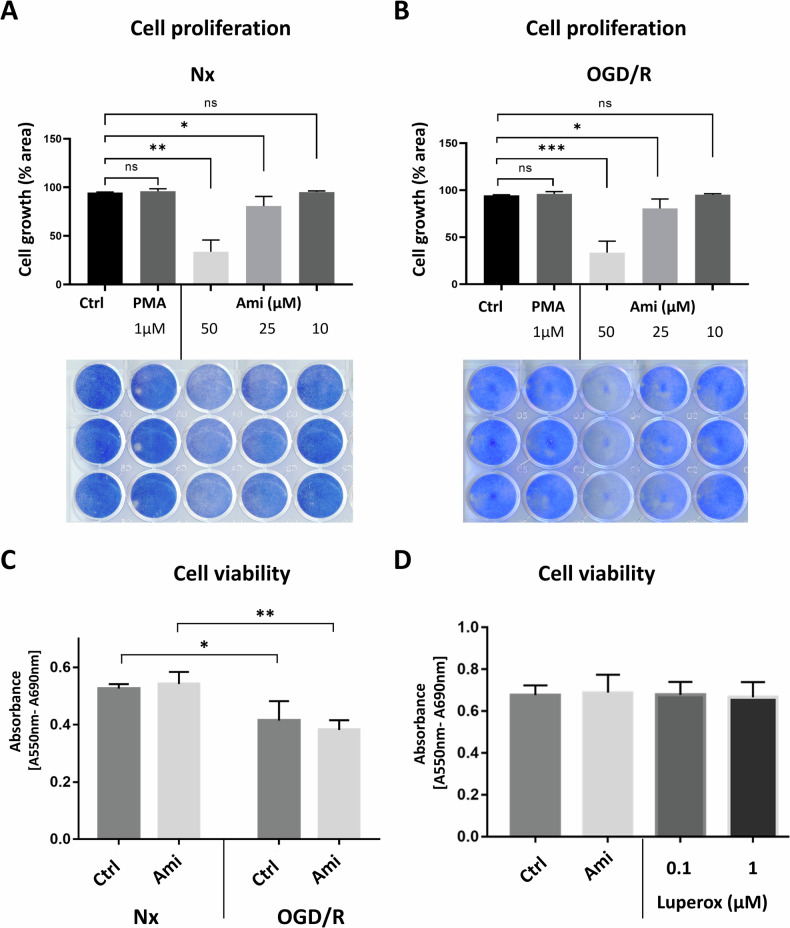
Amitriptyline reduces cerebral endothelial proliferation in normoxia and post-I/R without influencing cell survival. **A**, **B** Proliferation of cerebral microvascular endothelial hCMEC/D3 cells exposed to Nx (21% O_2_; in **A**) or OGD (1% O_2_) for 24 h followed by reoxygenation (Reox) for 48 h in full medium (OGD/R; in **B**), evaluated by Coomassie blue staining, after treatment with control medium (Ctrl), phorbol-12-myristate-13-acetate (PMA; 1 µM) or amitriptyline (Ami; 10–50 µM). **C**, **D** Cell viability, assessed by the 3-(4,5-dimethyl-2-thiazolyl)-2,5-diphenyl-2H-tetrazolium bromide (MTT) assay, of hCMEC/D3 cells exposed to Nx (21% O_2_) or OGD (1% O_2_) for 24 h followed by Reox for 24 h. Cells were treated with Ctrl or Ami (50 µM; in **C**) or Ctrl, Ami (50 µM) or Luperox^®^ (0.1 µM or 1 µM; in **D**). Plates were scanned and analyzed using ImageJ software. Data were processed as triplicates, of which mean values were formed. Results of 3 independent experiments are presented. Data are mean ± SD values. Evaluated by one-way or two-way ANOVA, as adequate, followed by Bonferroni tests. *p ≤ 0.05/**p ≤ 0.01 compared with corresponding Ctrl.

To further study the cellular impact of Ami, we analyzed the proliferation of endothelial hCMEC/D3 cells treated with the ASM activator PMA and the ASM inhibitor Ami using the colorimetric dye Coomassie blue. While ASM activation by PMA did not influence endothelial proliferation in Nx or OGD/R conditions, we noticed a dose-dependent growth inhibition after treatment with Ami in both conditions (Fig. 4A, B). At a dose of 50 µM, Ami reduced endothelial proliferation by ~60% in Nx and by ~80% in OGD/R. A possible explanation for the compromised growth was a drop of cell survival. 3-(4,5-dimethyl-2-thiazolyl)-2,5-diphenyl-2H-tetrazolium bromide (MTT) assays revealed that Ami did not influence cell viability regardless of the chosen experimental condition (Fig. 4C). Similarly, the chemical ROS inducer Luperox^®^ did not influence cell survival (Fig. 4D).

**Fig. 5:**
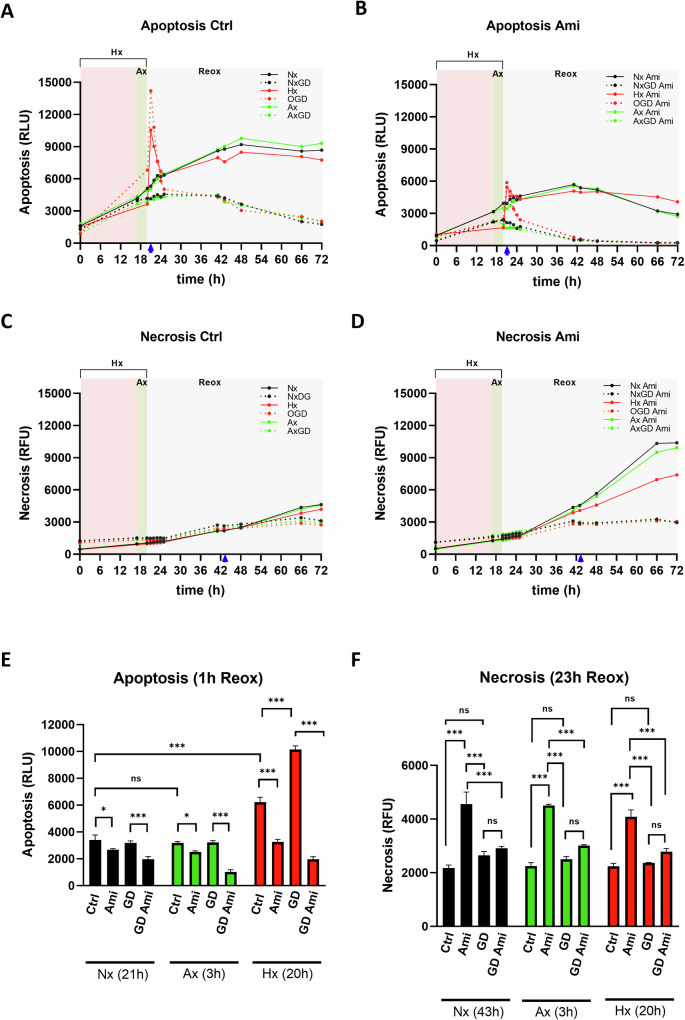
Amitriptyline protects cerebral endothelial cells against apoptotic cell death post-I/R. Live imaging of apoptotic and necrotic cell death using the RealTime-Glo^®^ Annexin V Apoptosis and Necrosis assay in cerebral microvascular endothelial hCMEC/D3 cells. Cells were grown in full medium (solid lines) or glucose deprivation (ΔG) medium (dashed lines), which were exposed to Nx (black lines), Hx for 20 h (red lines) or anoxia (Ax) for 3 h (green lines). Cell growth in ΔG medium exposed to Nx was labeled as GD (i.e., glucose deprivation), cell growth ΔG medium exposed to Hx as OGD (i.e., oxygen-glucose deprivation), and cell growth in ΔG medium exposed to Ax as AxGD (i.e., anoxia-glucose deprivation). Experiments were aligned for reoxygenation (Reox), which in all groups took place 20 h after the initiation of recording. Immediately after the baseline measurement at time point 0 h after the beginning of the recording, amitriptyline (Ami; 50 µM) was added to the cells. **A**, **B** Apoptotic death detected by the measurement of luminescence generated by the binding of phosphatidylserine (PS) on the outer cell membrane leaflet to a NanoLuc^®^ Binary Technology (NanoBiT^®^) luciferase. **C**, **D** Necrotic death detected by the measurement of fluorescence emitted by a non-membrane-permeable dye after cytosolic double-strand DNA binding. Blue arrows indicate time points that were further analyzed in the following. Quantitative analysis of (**E**) apoptotic cell death 1 h after Reox (i.e., after 21 h recording) and **F** necrotic cell death 23 h after Reox (i.e., after 43 h recording) for hCMEC/D3 cells exposed to all 12 experimental conditions outlined above. Data were processed as triplicates, of which mean values were formed. A representative result out of 5 independent experiments is presented. Data are mean ± SD values. Evaluated by multi-way ANOVA followed by Bonferroni tests. *p ≤ 0.05/**p ≤ 0.01/***p ≤ 0.001/ns not significant.

Next, we tested if treatment with Ami influenced cell death in the hCMEC/D3 model. To carry out continuous measurements in living cells, we seeded hCMEC/D3 cells in full medium or glucose deprivation (ΔG) medium and conducted a RealTime-Glo^®^ Annexin V Apoptosis and Necrosis assay in Nx, during and after hypoxia (Hx) (20 h duration) and during and after anoxia (Ax) (3 h duration) (Fig. 5A–D). Baseline measurements were performed in all groups at the start of the experiments. Immediately thereafter, Hx and/or glucose deprivation (GD) were induced. Ax was started 17 h after Hx. As such, experiments were aligned for reoxygenation (Reox), which in all groups took place 20 h after the initiation of recording. The analysis of apoptosis, which in this assay evaluates the binding of phosphatidylserine (PS) on the outer cell membrane leaflet to a NanoLuc^®^ Binary Technology (NanoBiT^®^) luciferase by use of annexin V fusion proteins, revealed that during the first 20 h of the study (until termination of Hx or Ax) apoptotic death was moderate and similar in all conditions except OGD, which revealed a 1.3-fold higher luminescence at the end of OGD compared to the other groups (Fig. 5A, B). In the absence of Ami, hCMEC/D3 cells exposed to Hx exhibited an apoptotic surge 1 h after Reox (i.e., after 21 h recording), which was more pronounced in the absence of glucose (i.e., OGD) than in the presence of glucose (i.e., Hx) (Fig. 5A). Of note, this apoptotic boost subsided 2 h after Reox (after 22 h recording). This boost was absent in cells exposed to 3 h Ax. After 24 h recording, all cells cultured in full medium developed a moderate apoptotic rise reaching a maximum at 50 h regardless of the oxygen level. In contrast, cells kept in GD showed instead a slow but constantly decreasing apoptotic signal. Remarkably, treatment with Ami protected against apoptotic cell death in all conditions examined (Fig. 5B, E). Particularly striking was the protective effect of Ami on the apoptotic surge 1 h after Hx (after 21 h recording), which was almost completely abolished by Ami regardless of the medium’s glucose content (Fig. 5B, E).

Hx, combined Hx and GD (i.e., OGD), Ax and combined Ax and GD (in the following abbreviated AxGD) did not induce significant necrotic death until 4 h after Reox (i.e., until 24 h recording), which in this assay evaluates the cytosolic accumulation of a non-membrane-permeable detection agent generating a fluorescence signal upon double-strand DNA binding (Fig. 5C, D). Necrotic death at this early time point was observed neither under control conditions (Ctrl) nor following exposure to Ami. At 22–24 h after Reox (i.e., after 42–44 h recording), however, significant necrosis was induced in cells cultured in full medium containing Ami, with the lowest level after Hx exposure (Fig. 5D, F). Notably, GD suppressed necrosis in Ami-treated cells (Fig. 5D, F).

### Ami reduces endoplasmic reticulum stress, stimulates autophagy, and stimulates antioxidant responses in normoxia and post-I/R

**Fig. 6:**
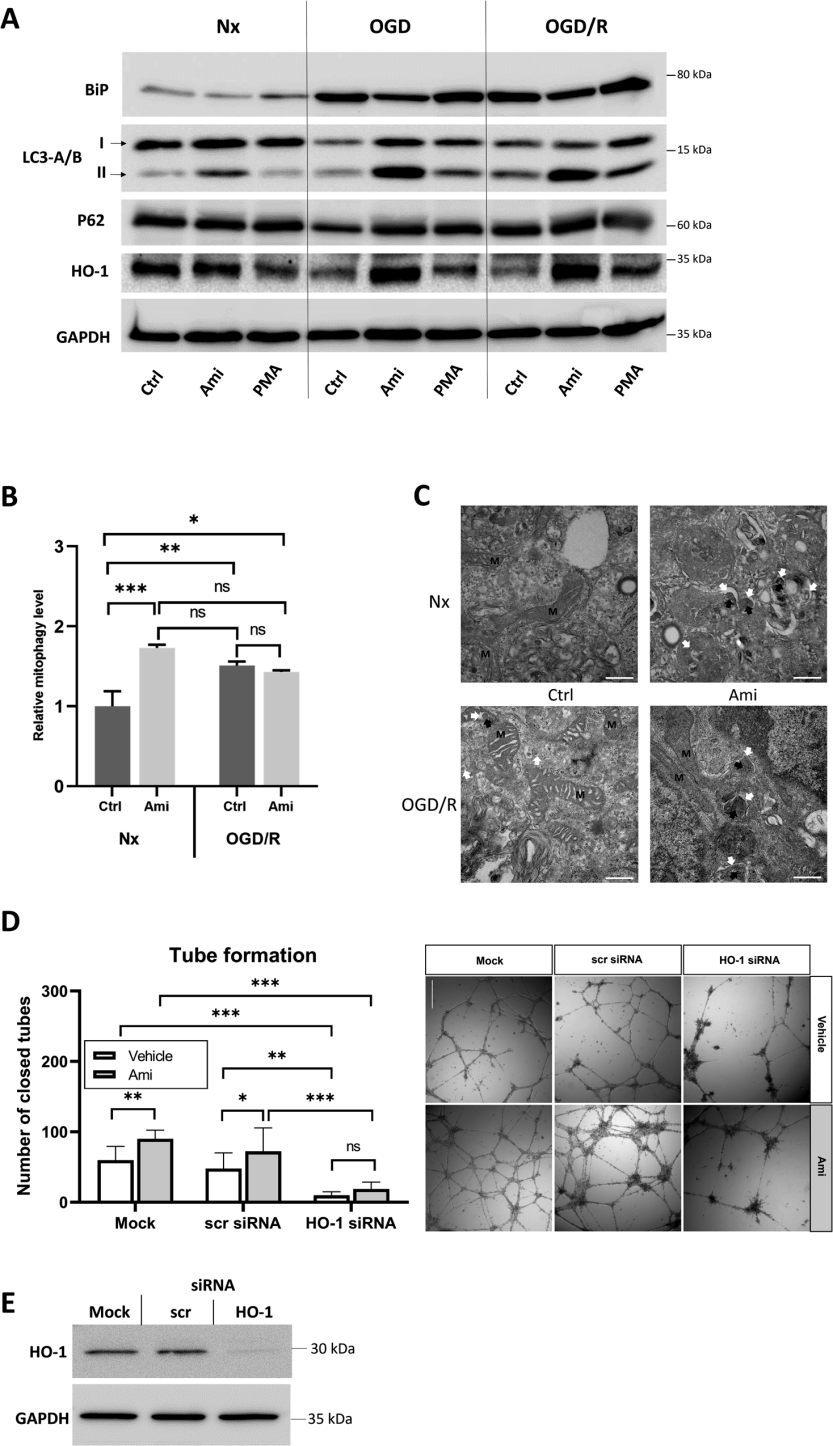
Amitriptyline reduces cerebral endothelial endoplasmic reticulum stress, stimulates autophagy/ mitophagy and heme oxygenase-1 (HO-1)-dependent antioxidant responses, which are instrumental for amitriptyline’s angiogenic effects. **A** Western blot analysis of protein lysates of cerebral microvascular endothelial hCMEC/D3 cells exposed to Nx, OGD for 24 h or OGD for 24 h followed by 3 h of reoxygenation (Reox; OGD/R), which were treated with control medium (Ctrl), the ASM inhibitor amitriptyline (Ami; 50 µM) or the ASM activator phorbol-12-myristate-13-acetate (PMA; 1 µM). Samples of 30 µg protein were loaded on each lane. The reproducibility of findings was confirmed in 3 independent experiments, of which a representative example was presented. GAPDH was used as loading control. **B** Mitophagy detection by flow cytometry in hCMEC/D3 cells. Cells were transfected with plasmid mKeima-Red-Mito-7 and treated as in (**A**). The expressed red fluorescent protein localizes in the mitochondrial inner membrane and shows a pH-dependent excitation switch from short wavelength (~440 nm) under neutral pH to long wavelength (~600 nm) under acidic pH that indicates the fusion of mitophagosomes with lysosomes. Samples were normalized to Nx Ctrl. Evaluated by two-way ANOVA followed by Bonferroni tests. *p ≤ 0.05/**p ≤ 0.01/***p ≤ 0.001. ns not significant (n = 3 independent samples/ group). **C** Transmission electron microscopy (TEM) images of hCMEC/D3 cells treated as in (**A**). Highlighted are intact mitochondria (M), autophagosomal structure (white arrow), and fragmented mitochondria (black arrow), Scale bar, 0.5 μm. n = 3 independent experiments. Representative images are shown for each condition. **D** Matrigel-based tube formation assay of hCMEC/D3 cells kept under mock conditions (Mock) or transfected with scrambled (scr) siRNA or HO-1 siRNA, which were treated with control medium (Ctrl) or Ami (50 µM). Representative samples are shown. Scale bar in representative tube formation photographs: 500 µm. **E** Western blot analysis of protein lysates of hCMEC/D3 cells kept under mock conditions or transfected with scrambled siRNA or HO-1 siRNA. GAPDH was used as loading control. Samples were analyzed using ImageJ software. Data are mean ± SD values. Evaluated by multi-way ANOVA followed by Bonferroni tests or LSD tests (**D**). *p ≤ 0.05/**p ≤ 0.01/***p ≤ 0.001. ns not significant (n = 3 independent samples/ group).

It is well known that Hx and Reox can induce ER stress and autophagy, and elicit antioxidant responses [17**–**19]. To evaluate these responses in the hCMEC/D3 model, we performed Western blot analysis of protein lysates of cells exposed to Nx, OGD (24 h) or OGD (24 h) followed by Reox (3 h; OGD/R), which were treated with the ASM inhibitor Ami or the ASM activator PMA (Fig. 6A). Compared to Nx controls which showed a low abundance of the ER stress sensor BiP [20] regardless of Ami or PMA administration, BiP abundance was elevated upon OGD or OGD/R. Ami, but not PMA reduced BiP abundance. The abundance of heme oxygenase-1 (HO-1), an ~30 kDa protein located in the ER membrane, which is a major mediator of antioxidant responses and promotes cell survival [21], was reduced by OGD and OGD/R and restored by Ami. Analysis of the autophagic marker protein LC3A/B-II indicated that Ami-induced autophagy activation more strongly in the OGD and OGD/R than in the Nx group. The ubiquitin-binding protein p62 was not influenced by OGD, OGD/R or Ami.

### Ami and I/R stimulate mitophagy

To investigate if the Ami-induced autophagy also eliminates dysfunctional mitochondria in the selective autophagic degradation process called mitophagy, we transfected hCMEC/D3 cells with mitophagy marker plasmid mKeima-Red-Mito-7 and exposed them to Nx or OGD (24 h) followed by Reox (3 h; OGD/R) (Fig. 6B). Subsequent flow cytometry analysis confirmed that Ami treatment induced a ~1.7-fold increase in mitophagy positive cells compared to Nx control. Notably, also OGD/R alone increased mitophagy by up to ~1.5-fold, but the combined treatment of Ami and OGD/R did not show any additive effect on the mitophagic level (~1.4-fold). Next, we conducted transmission electron microscopy (TEM) experiments in the hCMEC/D3 model to examine possible changes in the ultrastructure of the cells after Ami treatment (Fig. 6C). In line with the flow cytometry results, we detected the highest increase of autophagosome formation with incorporated mitochondrial fragments after Ami treatment in Nx. Again, OGD/R alone and the combined treatment with Ami did showed slightly less pronounced mitophagy.

### ASM inhibitor increases cerebral angiogenesis in a HO-1 antioxidant-dependent way

It has previously been shown that HO-1 is a major regulator of angiogenesis in embryonic development as well in animal models of I/R (ischemic stroke, myocardial infarction) and cancer [21, 22]. To evaluate the role of HO-1 in angiogenesis induced by the ASM inhibitor Ami, we induced a HO-1 knockdown in hCMEC/D3 cells by means of siRNA transfection and subsequently evaluated tube formation in response to Ami. HO-1 knockdown severely reduced the tube number to ~30% compared to scrambled siRNA control cells (Fig. 6D). Of note, Ami treatment was not able to rescue tube formation (Fig. 6D), underlining a key role of HO-1 in Ami-induced angiogenesis. HO-1 abundance was nearly completely depleted by HO-1 knockdown (Fig. 6E).

## Discussion

Using cerebral microvascular endothelial hCMEC/D3 cells exposed to OGD/R, an in vitro model of I/R injury, we showed that the ASM inhibitor Ami promotes angiogenesis in a ROS- and HO-1 antioxidant-dependent way. Increased ROS levels were noted in the intracellular compartment and, specifically, in the mitochondria of Ami-treated hCMEC/D3 cells, which were abolished by the ROS scavenger NAC, while a ROS inducer, Luperol®, mimicked Ami’s effect on cerebral angiogenesis. Ami-induced angiogenesis was not abrogated by scavenged mitochondrial ROS, suggesting that ROS produced by other cellular compartments also account for the effect on tube formation. Increased ROS formation in response to Ami was also found in vivo in the vascular compartment of mice exposed to transient intraluminal MCAO, a widely used ischemic stroke model. Importantly, angiogenesis was not induced at the expense of compromised cerebral endothelial survival. Thus, ASM inhibition was found to induce a complex network of protective cellular responses, which involved reduced cell proliferation, reduced mitochondrial energy metabolism, reduced ER stress, increased autophagy, and increased antioxidant responses, which protected against I/R injury and prevented apoptotic cell death. Importantly, ROS formation was not increased by the ASM inhibitors Ami or fluoxetine in the brain parenchyma post-I/R of MCAO mice. Our study for the first time identified the metabolic responses that contribute to angiogenesis and brain remodeling by ASM inhibitors post-I/R.

We have previously described that the antidepressants Ami, fluoxetine, and desipramine promote cerebral angiogenesis in vitro and in vivo post-I/R in an ASM-dependent way via the release of small extracellular vesicles (EVs) from endothelial cells [10]. ASM knockdown in cerebral microvascular endothelial hCMEC/D3 cells transfected with sphingomyelinase phosphodiesterase-1 (*Smpd-1*; i.e., the gene encoding ASM) siRNA abolished the angiogenic effects of Ami similar to *Smpd-1*^-/-^ in mice exposed to transient intraluminal MCAO [10]. The delivery of EVs obtained from hCMEC/D3 cells treated with Ami-induced angiogenesis equally as Ami [10]. In the brains of MCAO mice, the delivery of Ami restored blood-brain barrier integrity, reduced leukocyte infiltrates, and increased neuronal survival post-I/R [10]. These studies established a neurorestorative function of ASM inhibitors. The cellular mechanisms responsible for inducing angiogenesis had largely been unknown. It has already been reported that ASM inhibitors including Ami can trigger oxidative stress [13].

The vascular endothelium is a primary site of ROS generation post-I/R and major target of ROS-dependent cell injury [7]. When successfully coping with I/R injury, brain microvessels are able to reestablish their barrier functions and sprout, thus contributing to successful stroke recovery [8]. Yet, excessive ROS formation exacerbates cell damage in the brain parenchyma post-I/R [23, 24], causing damage to lipids, proteins, and DNA [6], whereas ROS inhibition or scavenging protects against I/R injury [25, 26]. ASM inhibitors are frequently prescribed for the treatment of post-stroke depressive disorder [27]. Hence, understanding the consequences of ASM inhibitor-induced ROS formation for the survival and angiogenic sprouting of cerebral endothelial cells has pivotal clinical importance under conditions of I/R injury. In the present study, we found that the ASM inhibitor Ami increased intracellular and, more specifically, mitochondrial ROS levels in cerebral endothelial hCMEC/D3 cells exposed to OGD/R and also increased lipid peroxidation in the vascular compartment of mice exposed to transient MCAO. Despite this increased ROS formation, cerebral endothelial cells treated with Ami were protected against I/R, exhibiting reduced apoptotic cell death. The effects of Ami on cell survival during oxidative stress are cell type-dependent. In cancer cells, increased ROS formation induced by Ami was found to suppress cellular antioxidant responses, inducing irreversible mitochondrial damage and apoptotic cell death [28]. The authors suggested that Ami might be considered as oxidation therapy in cancer.

Compared with cancer cells, cerebral microvascular endothelial hCMEC/D3 cells were highly resistant to Ami, even under conditions of I/R. As likely reason, mitochondrial respiration (including ATP-linked respiration and spare respiratory capacity), which was increased by I/R, was potently reduced by Ami, as was MMP, another measure of mitochondrial ETC activity [16]. Endothelial cells generate more than 80% of their ATP through glycolysis [29], which explains their low basal mitochondrial respiration compared to other cell types. Using hCMEC/D3 cells, we confirmed that basal mitochondrial respiration was low in cerebral endothelial cells compared with primary neurons and astrocytes. Mitochondrial ETC activity, particularly of complexes I and III, is known to predispose to ROS formation [21]. Hence, the reduced mitochondrial activity represented an efficient mean enabling cell survival. The question remains how Ami increased mitochondrial ROS formation despite reduced respiration activity. A putative mechanism may be electron leakage from a disturbed mitochondrial complex III, which has previously been reported following ROS formation under Hx/R conditions [30].

In this study, I/R elicited a clear-defined ER stress response in cerebral endothelial hCMEC/D3 cells, indicated by the stress sensor BiP [20], which was elevated upon OGD or OGD/R. In contrast, HO-1, which is a key antioxidant that is primarily located in the ER membrane facing the cytosol [31], was reduced by OGD or OGD/R, and restored by Ami. HO-1 expression is orchestrated by the transcription factor nuclear factor erythroid-2-related factor (NRF-2), which gets stabilized via two alternative pathways: by oxidation via ROS or by phosphorylation via activated protein kinase RNA-like ER kinase (PERK). PERK is a major mediator of the unfolded protein response (UPR) pathway that is activated by ER stress [32, 33]. NRF-2 pathway activation prevents apoptosis via a variety of proteins including Bcl-2, superoxide dismutase (SOD), and notably HO-1 [33, 34]. HO-1 has antiapoptotic effects mainly by its enzymatic reaction products biliverdin-IXα, iron, and carbon monoxide [35]. In the present study, HO-1 was instrumental for Ami’s ability to induce tube formation in cerebral endothelial hCMEC/D3 cells. It has previously been shown in a variety of I/R injury and cancer models that HO-1 controls angiogenesis partly by increasing vascular endothelial growth factor (VEGF) formation [21, 22]. In hCMEC/D3 cells, angiogenesis was almost completely abolished by HO-1 knockdown.

As reported previously for primary neurons [36], Ami-induced a potent autophagy activation, evidenced by the autophagic marker protein LC3A/B-II, in endothelial hCMEC/D3 cells, which was more pronounced in OGD and OGD/R than in Nx groups. ROS is a known trigger for the expression of autophagic proteins through the activation of transcription factors including NRF-2, HIF-1, and p53 [37]. The NRF-2 target HO-1 seems to play a critical role for autophagy induction in endothelial cells [38]. It has been reported that ROS can oxidize the autophagy-related protease ATG4 that inhibits LC3B delipidation, thereby ensuring continuous autophagosome formation [39]. The promotion of autophagy likely contributed to cell survival [38]. Since autophagy activation is known to induce a cell cycle arrest in the G2 mitosis phase [40, 41], it could explain the inhibition of cell proliferation by Ami that occurred without a significant drop of energy state. That Ami protects against cell death has previously been shown in mouse cardiomyocytes by Daia and coworkers [42]. In that earlier study, Ami prevented Hx/R-induced apoptotic cell death by activating tropomyosin receptor kinase A receptor (TrkA) and Akt signaling [42]. Here, we also found Ami-induced mitochondrial autophagy, which is another cell protective mechanism that copes with damaged mitochondria post-I/R [43]. In the OGD studies, we made use of moderate hypoxia (1% O_2_), which is widely used in the I/R field. 1% O_2_ corresponds to oxygen levels in the ischemic penumbra, but it hardly reflects the severe oxygen depletion in the ischemic infarct core (<0.1% O_2_) [44]. Since a former study in HUVEC cells reported an apoptotic surge after a short period of Ax followed by reoxygenation [45], we chose the same settings in the hCMEC/D3 model but found no excess apoptotic nor necrotic cell death compared to normoxic control conditions. This observation indicates a high cellular robustness of the cerebral endothelium even in severe oxygen depletion, which is supported by an earlier study on pulmonary arterial endothelial cells, in which endothelial cells survived much longer periods of anoxia (up to 18 h) without significant changes of cellular integrity [46]. In cerebral endothelial hCMEC/D3 cells exposed to Ax or AxGD, Ami did not induce apoptotic or necrotic cell injury. These data exemplify the absence of detrimental effects related to the ASM inhibitor across a broad variety of injury severities.

Blood glucose level is strictly regulated in mammals, and cerebral hypoglycemia is a major threat for neurons in the human brain [47]. Yet, several in vitro studies showed that glucose deprivation alone does not deplete intracellular ATP level, at least not in non-neuronal cells [48, 49]. In the absence of glucose, mitochondria can consume protons for ATP production, which may provoke lysosomal alkalinization and induce necrosis independent of the energy level in cancer cells [50]. A previous study claimed that the UPR protein ATF-4 plays a role in the induction of necrosis by glucose deprivation [51]. Although we observed pronounced ER stress in response to OGD, our data differed from these earlier studies since hCMEC/D3 cells exposed to glucose deprivation in general revealed the lowest apoptotic and necrotic death rates. A critical exception was the early Reox phase, in which the Hx-induced apoptotic boost was amplified by additional glucose deprivation. Interestingly, the apoptotic boost in response to OGD/R was markedly attenuated by Ami. There is a large body of evidence that illustrates the harmful effects of high glucose in mammalian cells, e.g., via mitochondrial ROS formation [52] and protein kinase-C activation [53], which in turn produces additional ROS via NADPH oxidase [54].

Taken together, this study was able to reveal a hitherto unknown dual mode of action of the ASM inhibitor amitriptyline, that is, the promotion of angiogenesis in a ROS- and HO-1-dependent way, which is accompanied by the reregulation of mitochondrial energy metabolism, attenuation of ER stress, promotion of autophagy/ mitophagy, promotion of antioxidant responses, and inhibition of apoptosis post-I/R. These joint actions may provide the key for the previously reported successful brain remodeling induced by ASM inhibitors after transient MCAO. Representing a pathway that decisively controls cell signaling via modification of cell membrane microdomains, the ASM–ceramide system represents a promising target for restorative treatments in I/R injuries.

## Materials and methods

### Cell lines and cell culture

Human cerebral microvascular endothelial hCMEC/D3 cells were cultivated from passage 28–35 in Growth Basal Medium-2 (EBM-2, Lonza, Basel, Schweiz) supplemented with 5% fetal bovine serum (FBS, Life Technologies, Carlsbad, CA, U.S.A.), 100 U/ml penicillin/streptomycin (Life Technologies), 1.4 µM hydrocortisone (Sigma–Aldrich, Saint Louis, U.S.A.), 5 µg/ml ascorbic acid (Sigma–Aldrich), 1% chemically defined lipid concentrate (Life Technologies), 10 mM HEPES (Life Technologies) and 1 ng/ml basic fibroblast growth factor (bFGF, Sigma–Aldrich) in a humidified atmosphere at 37 °C containing 21% O_2_ and 5% CO_2_. Oxygen-glucose deprivation (OGD) was induced in a hypoxia chamber (1% O_2_, Toepffer Lab Systems, Göppingen, Germany) with glucose-free medium (Life Technologies) supplemented as described before. Anoxia was induced by incubating the cells in an AnaeroBox^®^ (<0.1% O_2_, #AB0035L, Thermo Fisher Scientific, Waltham, MA, U.S.A.). Primary astrocyte and neuron cultures were prepared as described before [55, 56]. Human umbilical vein endothelial cells (HUVEC) were cultured in endothelial cell growth medium (ECGM, PromoCell) containing 100 U/ml penicillin/streptomycin (Life Technologies) and growth medium supplemental mix (PromoCell). Human neuroblastoma (SH-SY5Y) cells were cultured in Dulbecco’s modified Eagle medium (DMEM, Gibco, Thermo Fischer Scientific) supplemented with 10% fetal bovine serum (FBS), 100 U/ml penicillin/ streptomycin (Life Technologies). All experiments were performed with mycoplasma-free cells.

### Tube formation assay

To analyze the formation of capillary-like tubular structures, 60 µl Matrigel (Corning, NY, U.S.A.) were pipetted into 96-well plates. The gel solidified at 37 °C for 30 min. 3 × 10^4^ cells were seeded and treated, as outlined in the main text. 20 h later, images were taken using a digital inverted microscope with 2× magnification (AMG EVOS fl; Advanced Microscopy Group, Bothell, WA, U.S.A.). Closed tubes were counted using Image J (NIH) in each well. Experiments were done in triplicates, of which mean values were formed.

### Endothelial viability assay

2 × 10^4^ hCMEC/D3 cells were seeded in 96-well plates and treated as described in the main text. After 24 h, cells were incubated with 0.5 mg/ml 3-(4,5-dimethyl-2-thiazolyl)-2,5-diphenyl-2H-tetrazolium bromide (MTT; Biomol, Hamburg, Germany) for 2 h. Cells were fixed with DMSO (Sigma–Aldrich). Light absorbance was measured using a microplate absorbance reader (iMark^®^; Bio-Rad Laboratories, Hercules, CA, U.S.A.) at 570 nm wavelength. Samples were analyzed in triplicates, of which mean values were formed.

### Endothelial proliferation assay

5 × 10^4^ cells were seeded in 24-well plates and treated, as described in the main text. Cells exposed to OGD for 24 h were reoxygenated and incubated for additional 48 h. Finally, the medium was decanted and cells were fixed in 0.1 M phosphate-buffered saline (PBS) containing 2% paraformaldehyde (PFA) for 20 min followed by 10 min incubation with 70% ethanol in PBS and staining in Coomassie blue. After continuous washing for 1–2 h, the plates were dried and scanned for area density analysis using Image J software.

### Sphingomyelinase activity assay

Cells were lysed in 250 mM sodium acetate buffer (pH 5.0) containing 1% NP-40 detergent (Fluka BioChemika, Morristown, NJ, U.S.A.), and cellular membrane integrity was subsequently disrupted with a sonicator. After centrifugation for 5 min at 300 × *g* at 4 °C, supernatants were collected. Lysates were adjusted to a specific protein concentration and incubated with 100 pmol BODIPY-labeled sphingomyelin (Thermo Fisher Scientific) in 250 mM sodium acetate (pH 5.0) and 0.1% NP-40 for 1 h at 37 °C. Chloroform:methanol (2:1) was added, and samples were vortexed and centrifuged for 5 min at 15,000 × *g* to achieve phase separation. The lower phase was collected and concentrated in a vacuum centrifuge (SPC111V, Thermo Fisher Scientific) for 45 min at 37 °C. Lipids were dissolved in 20 µl chloroform:methanol (2:1) and spotted onto thin layer chromatography (TLC) plates (Macherey Nagel, Düren, Germany). The TLC run was performed with chloroform:methanol (80:20). TLC plates were analyzed with a Typhoon FLA 9500 scanner (Cytiva, Marlborough, MA, U.S.A.), and lipid spots were quantified with Image Quant (Cytiva).

### Cellular and mitochondrial ROS detection

Oxidative stress measurements were performed using CellROX^®^ Green (Thermo Fisher Scientific) for the detection of cellular ROS and MitoSOX^®^ Red (MedChemExpress, Monmouth Junction, NJ, U.S.A.) for the detection of mitochondrial ROS following the manufacturers’ protocols.

### Mito stress assay

Mitochondrial respiration was measured using Seahorse XF Cell Mito stress assay, (103344-100; Agilent, Santa Clara, CA, U.S.A). All procedures were performed according to instructions provided by the manufacturer. Subsequently, basal OCR and ECAR were measured prior to and after sequential injection of 5, 1.25, and 5 µM oligomycin, FCCP, and Rot/AA, respectively, using a Seahorse XFe24 analyzer (Agilent, Santa Clara, CA, U.S.A.). After measurement, cells were fixed using 0.4% PFA and stained with Hoechst 33342 (H3570; Thermo Fisher Scientific). Images were acquired from Hoechst-stained nuclei using AMG EVOS FL MICROSCOPY (Thermo Fisher Scientific) and nuclei were counted using Fiji Image J. All parameters were normalized to the quantified nuclei using Wave desktop 2.4 software (Agilent). Mito stress reports were generated in wave software, and basal respiration, ATP-linked respiration, and spare respiratory capacity values were extracted.

### Mitochondrial membrane potential analysis

To evaluate mitochondrial membrane potential (MMP; Ϫѱ), 5 nM tetramethylrhodamine-methyl ester perchlorate (TMRM) was added to treated cell cultures. After 24 h incubation, live cell confocal imaging was performed using a Leica TCS SP8 fully automated epifluorescence confocal microscope (Zeiss, Wetzlar, Germany). TMRM fluorescent intensity was recorded before and after injecting 5 µM carbonyl cyanide m-chlorophenylhydrazone (CCCP). CCCP is a mitochondrial uncoupler that abolishes MMP, thereby providing an insight into the probable background fluorescent.

### Mitophagy detection by flow cytometry

For transient transfection, 1 µg of plasmid mKeima-Red-Mito-7 (# 56018, Addgene) was transfected into 1 × 10^5^ pre-seeded hCMEC/D3 cells using Lipofectamin^TM^ 3000 (Thermo Fisher Scientific). A red laser (633 nm) coupled with a 660/ 20 nm bandpass filter (APC channel) was employed to detect the excitation switch caused by a drop of pH as the last step of autophagy [57]. At least 20.000 cells per sample were analyzed on a LSRII (BD Biosciences, Franklin Lakes, NJ, USA) using FACS DIVA software.

### Transmission electron microscopy (TEM)

HMEC/D3 cells were cultured in glass-bottom dishes (µ-Dish 35 mm high Grid-500; Ibidi GmbH, Gräfelfing, Germany). At the time point of analysis, the cells were fixed using 4% formaldehyde + 2.5% glutaraldehyde dissolved in 0.1 M PHEM buffer, overnight, at 4 °C. All following steps of the sample preparation, including post-fixation with osmium tetroxide, contrasting with uranyl acetate, dehydration in an ascending ethanol row, and EPON^TM^ (Poly/Bed 812, Polysciences Inc., Warrington USA) infiltration were performed using a laboratory microwave oven (PELCO BioWave Pro+, Ted Pella, Redding, USA). A table containing the microwave protocol details can be found in the supplemental information. The resin polymerization then took place at 60 °C for 96 h. After subsequent glass-bottom removal and release from the dish the resin disks were trimmed to areas of interest. Ultrathin sections of 60 nm in diameter were cut using an ultramicrotome (EM UC7, Leica Microsystems, Wetzlar, Germany) equipped with a diamond knife (ultra 35°, Diatome, Nidau, Switzerland). Sections were collected on 200 mesh hexagonal thin-bar TEM copper grids (Science Services, Munich, Germany) and post-contrasted with 4% aqueous uranyl acetate and Reynold’s lead citrate solution using an automated contrasting instrument (EM AC20, Leica Microsystems, Wetzlar, Germany). Image acquisition was performed with a JEM 1400Plus TEM (JEOL Ltd., Tokyo, Japan), operating at 120 kV and equipped with a 4096 × 4096 pixels CMOS camera (TemCam-F416, TVIPS, Gauting, Germany) using SerialEM v3.8.0. Resulting 16-bit TIFF images were processed in Fiji v1.53c.

### Real-time apoptosis and necrosis assay

To measure continuously apoptotic and necrotic events, we used the RealTime-Glo^®^ Annexin V Apoptosis and Necrosis assay (#A1011; Promega, MD, U.S.A.) following the manufacturer’s protocol. Briefly, 2 × 10^4^ cells/ well were seeded in a black 96-well plate. Addition of the detection reagents to the cells 18 h later defines time point t = 0. At each chosen time point, green fluorescence intensity (λex /λem = 485 nm/520 nm) and luminescence intensity was measured with the fluorescence microplate reader FLx800 (Biotek, Winooski, VT, U.S.A.) over a time period of 96 h to quantify apoptosis and necrosis, respectively.

### siRNA transfection

For siRNA transfection, consumables were purchased from OriGene Technologies (Rockville, MD, U.S.A.). Following the manufacturer’s instructions, cells were transfected with 45 nM of either scrambled siRNA or a mix of three different siRNAs (15 nM each) against HO-1 mRNA (NM_002133) using siTran 2.0^®^ transfection reagent.

### Antibodies and reagents

Anti-BiP (#3177), Anti-LC3A/B (#4108) and Anti-GAPDH (#5174) antibodies were purchased from Cell Signaling Technologies (Danvers, MA, U.S.A.). Anti-p62 (18420-1-AP) antibody was from Proteintech (Planegg-Martinsried, Germany) and Anti-HO-1 (ab68477) antibody was from Abcam (Cambridge, U.K.).

### Gel electrophoresis and Western blotting

Whole-cell extracts were prepared using radioimmunoprecipitation (RIPA) buffer. Proteins were separated by sodium dodecyl sulfate (SDS)-polyacrylamide gel electrophoresis (PAGE) using a 7.5% polyacrylamide gel and subsequently transferred onto a polyvinylidene fluoride (PVDF) membrane. 5% skimmed milk in tris-buffered saline (TBS; 50 mM Tris/HCl, 150 mM NaCl; pH 7.2) containing 0.5% Tween-20 (TBS-T) were used to block the membrane against unspecific antibody binding. Incubation with antibodies was performed according to the manufacturers’ recommendations. Secondary antibodies were detected using an enhanced chemoluminescence (ECL) kit (34095; Thermo Fisher Scientific) via a ChemoCam Imager (INTAS Science Imaging, Göttingen, Germany). Protein abundance was quantified by densitometry of protein bands using the software IMAGEJ. After normalization to each loading control, samples were compared to the untreated control sample (set to 1). The full-length western blots can be found in the supplementary file titled “Uncropped Western blots”.

### Transient middle cerebral artery occlusion (MCAO)

All experiments were performed with local government approval (Bezirksregierung Düsseldorf) in accordance with EU guidelines (Directive 2010/63/EU) for the care and use of laboratory animals, STAIR, STEPS, and ARRIVE guidelines. The experimenters were fully blinded at all stages of the study by another researcher preparing the vehicle and drug solutions. These solutions received dummy names (A, B, and C), which were unblinded after the termination of the study. Focal cerebral ischemia was induced in male C57BL6/j mice (8–10 weeks; 22–25 g; Harlan Laboratories, Darmstadt, Germany) anesthetized with 1.0–1.5% isoflurane (30% O_2_, remainder N_2_O) by 30 min left-sided intraluminal MCAO [30]. Rectal temperature was maintained between 36.5 and 37.0 °C using a feedback-controlled heating system (Fluovac; Harvard apparatus, Holliston, MA, U.S.A.). Cerebral blood flow was recorded by laser Doppler flow (LDF) measurement using a flexible probe attached to the skull overlying the middle cerebral artery territory core. The left common and external carotid arteries were isolated and ligated, and the internal carotid artery was temporarily clipped. A silicon-coated nylon monofilament (Doccol Corp., Sharon, MA, U.S.A.) was introduced through a small incision into the common carotid artery and advanced to the carotid bifurcation for MCAO. Reperfusion was initiated by monofilament removal. Wounds were carefully sutured. Vehicle, amitriptyline (12 mg/kg; Sigma–Aldrich, Deisenhofen, Germany) or fluoxetine (10 mg/kg, Sigma–Aldrich) was administered starting immediately after MCAO. For analgesia, buprenorphine (0.1 mg/kg; Reckitt Benckiser, Slough, UK) was administered before MCAO. For antiinflammation, animals received carprofen (4 mg/kg; Bayer Vital, Leverkusen, Germany) injections after MCAO. At 24 h post-MCAO, mice were sacrificed by transcardiac perfusion with PBS. Sample size planning was conducted with G*Power version 3.1.7 software of the University of Düsseldorf, Germany. According to pre-defined exclusion criteria, mice suffering from respiratory abnormalities or from severe nurturing handicaps resulting in a weight loss >20% should be removed from the study. None of the mice used for this study met these criteria.

### Measurement of oxidative stress in vivo

Oxidative stress was evaluated using the thiobarbituric acid reactive substances (TBARS) assay kit (R&D Systems, Minneapolis, U.S.A.). Ischemic brain tissue samples were lysed with NP-40 buffer. Blood samples were collected in heparin tubes and centrifuged at 1000 × *g*. For acidification, samples were incubated with TBA reagent for 15 min at room temperature and thereafter centrifuged at 12,000 × *g*. The supernatants were treated with TBA reagent. After incubation for 2 h at 50 °C, the optical density was measured at 532 nm.

### Statistical analysis

All experimental results were confirmed in at least 3 independent experiments. Bar graphs represent mean values plus standard deviations (SDs). In box plots, median values ± interquartile ranges (IQRs) with minimum and maximum values as whiskers are shown. For multiple group comparisons, one-way analysis of variance (ANOVA) or two-way ANOVA was used, as appropriate, followed by post hoc Bonferroni or LSD tests. Statistical tests were performed using GraphPad Prism 7 (La Jolla, CA, U.S.A.). P values ≤ 0.05 were considered significant.

## Supplementary information

Supplementary data

## Data Availability

The data that support the findings of this study are available from the corresponding authors upon reasonable request.
