# Supplementary material for: Acid sphingomyelinase inhibition induces cerebral angiogenesis post-ischemia/reperfusion in an oxidative stress-dependent way and promotes endothelial survival by regulating mitochondrial metabolism
Source: Cell Death Dis. 2024 Sep 4;15(9):650. doi: 10.1038/s41419-024-06935-9 (PMC11374893; doi:10.1038/s41419-024-06935-9)

Supplementary data

Acid sphingomyelinase inhibition induces cerebral angiogenesis post-ischemia/reperfusion in an oxidative stress-dependent way and promotes endothelial survival by regulating mitochondrial metabolism

Ayan Mohamud Yusuf[1,*], Mina Borbor[1,*], Tanja Hussner[1,*], Carolin Weghs[1], Britta Kaltwasser[1], Matthias Pilath-Eilers[1], Bernd Walkenfort[2], Richard Kolesnick[3], Erich Gulbins[4], Dirk M. Hermann[1,*,#] and Ulf Brockmeier[1,*,#]

[1]Department of Neurology, University Hospital Essen, University of Duisburg-Essen, Germany; [2]Imaging Center Essen (Electron Microscopy), University Hospital Essen, University Duisburg-Essen, Germany [3]Memorial Sloan Kettering Cancer Center, New York, New York, U.S.A.; [4]Department of Molecular Biology, University Hospital Essen, University of Duisburg-Essen, Germany

Table S1. TEM: Microwave embedding protocols

Step#	Description	Solution Change (Yes / No)	User Prompt (on /off)	Time (Hr:min:sec)	Power (Watts)	Vaccum (on / off)	Steady Temp temp (°C)
	Washing						
1	wash with 0.1 M PHEM	Yes	on	0:00:40	250	off	20
2	wash with 0.1 M PHEM	Yes	on	0:00:40	250	off	20
3	wash with 0.1 M PHEM	Yes	on	0:00:40	250	off	20
4	wash with 0.1 M PHEM	Yes	on	0:00:40	250	off	20
	2. Fixation and Osmification						
5	1% OsO4 in 0.1 M PHEM	Yes	on	0:02:00	100	vac on	20
6	1% OsO4 in 0.1 M PHEM	No	off	0:02:00	0	vac on	20
7	1% OsO4 in 0.1 M PHEM	No	off	0:02:00	100	vac on	20
8	1% OsO4 in 0.1 M PHEM	No	off	0:02:00	0	vac on	20
9	1% OsO4 in 0.1 M PHEM	No	off	0:02:00	100	vac on	20
10	1% OsO4 in 0.1 M PHEM	No	off	0:02:00	0	vac on	20
11	1% OsO4 in 0.1 M PHEM	No	off	0:02:00	100	vac on	20
12	1% OsO4 in 0.1 M PHEM	No	off	0:02:00	0	vac on	20
	Washing						
13	wash with 0.1 M PHEM	Yes	on	0:00:40	250	off	20
14	wash with 0.1 M PHEM	Yes	on	0:00:40	250	off	20
15	wash with H_2O	Yes	on	0:00:40	250	off	20
16	wash with H_2O	Yes	on	0:00:40	250	off	20
	Contrasting						
17	1% UA in H_2O	Yes	on	0:02:00	100	vac on	20
18	1% UA in H_2O	No	off	0:02:00	0	vac on	20
19	1% UA in H_2O	No	off	0:02:00	100	vac on	20
20	1% UA in H_2O	No	off	0:02:00	0	vac on	20
21	1% UA in H_2O	No	off	0:02:00	100	vac on	20
22	1% UA in H_2O	No	off	0:02:00	0	vac on	20
23	1% UA in H_2O	No	off	0:02:00	100	vac on	20
24	1% UA in H_2O	No	off	0:02:00	0	vac on	20
	Washing						
25	wash with H_2O	Yes	on	0:00:40	250	off	20
26	wash with H_2O	Yes	on	0:00:40	250	off	20
27	wash with H_2O	Yes	on	0:00:40	250	off	20
28	wash with H_2O	Yes	on	0:00:40	250	off	20
29	wash with H_2O	Yes	on	0:00:40	250	off	20
	Dehydration						
30	30% EtOH	Yes	on	0:00:40	150	off	20
31	50% EtOH	Yes	on	0:00:40	150	off	20
32	70% EtOH	Yes	on	0:00:40	150	off	20
33	80% EtOH	Yes	on	0:00:40	150	off	20
34	96% EtOH	Yes	on	0:00:40	150	off	20
35	100% EtOH dry	Yes	on	0:00:40	150	off	20
36	100% EtOH dry	Yes	on	0:00:40	150	off	20
37	Acetone	Yes	on	0:00:40	150	off	20
38	Acetone	Yes	on	0:00:40	150	off	20
39	Acetone	Yes	on	0:00:40	150	off	20
	Infiltration						
40	Epon:Acetone 1 : 3	Yes	on	0:03:00	250	vac on	20
41	Epon:Acetone 1 : 1	Yes	on	0:03:00	250	vac on	20
42	Epon:Acetone 3 : 1	Yes	on	0:03:00	250	vac on	20
43	Epon pure	Yes	on	0:03:00	250	vac on	20
44	Epon pure	Yes	on	0:03:00	250	vac on	20
	Polymerisation for 96 hr at 60°C						
UA	Uranyl Acetate						
EtOH	Ethanol						
vac	Vacuum						

Uncropped western blots

Fig.6A

Fig.6A- BiP

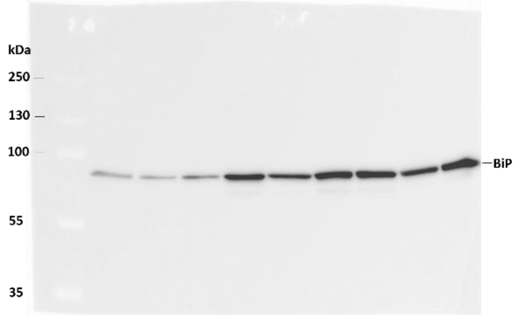

Fig.6A- LC3-A/B

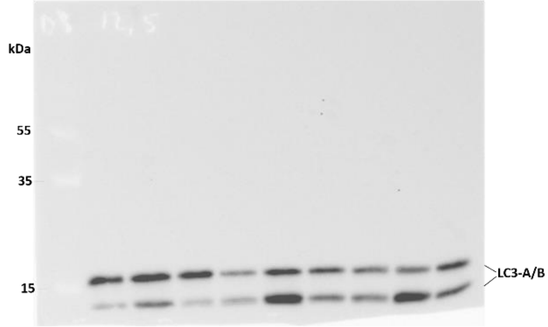

Fig.6A - P62

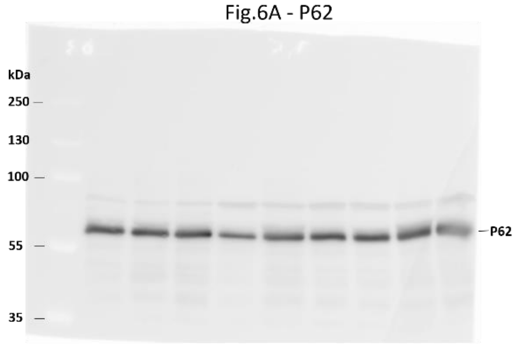

Fig.6A – HO-1

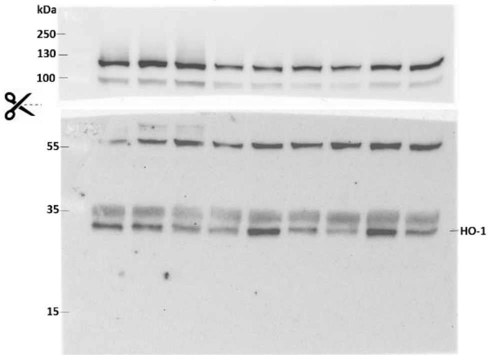

Fig.6A – GAPDH

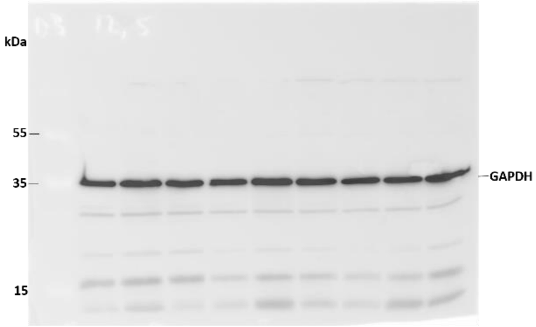

Fig.6C

Fig.6C – HO-1

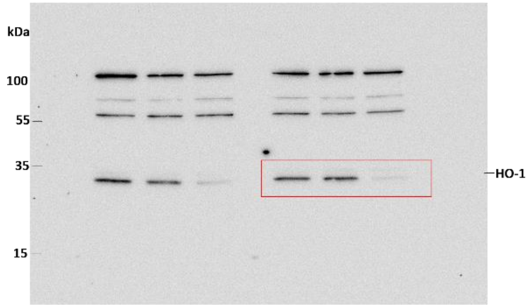

Fig.6C – GAPDH

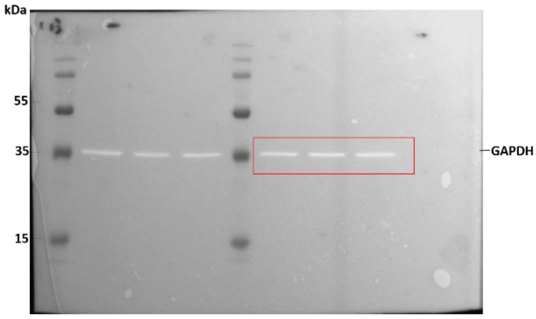

Supplement: Supplementary file 1 — Supplementary data [file 41419_2024_6935_MOESM1_ESM.pdf]
